# Supplementary material for: Resolving the full spectrum of human genome variation using Linked-Reads
Source: Genome Res. 2019 Apr;29(4):635–45. doi: 10.1101/gr.234443.118 (PMC6442396; doi:10.1101/gr.234443.118)
Supplement: Supplemental Material [file supp_gr.234443.118_Supplemental_longranger-2.2.zip › longranger-2.2.tar.bz2!/lr2.2-public/lib/stan/cmdstan/stan/doc/api/html/dir_e7242831226ee912bf5f374de664968d.html]

Stan: src/stan/mcmc Directory Reference


|  |  |
| --- | --- |
|  | Stan  2.5.0  probability, sampling & optimization |


- Main Page
- Related Pages
- Namespaces
- Classes
- Files

All Classes Namespaces Files Functions Variables Typedefs Enumerations Enumerator Friends Macros Pages

- src
- stan
- mcmc

mcmc Directory Reference

|  |  |
| --- | --- |
| Directories | |
| directory | hmc |
|  | |

|  |  |
| --- | --- |
| Files | |
| file | base\_adaptation.hpp [code] |
|  | |
| file | base\_adapter.hpp [code] |
|  | |
| file | base\_mcmc.hpp [code] |
|  | |
| file | chains.hpp [code] |
|  | |
| file | covar\_adaptation.hpp [code] |
|  | |
| file | fixed\_param\_sampler.hpp [code] |
|  | |
| file | sample.hpp [code] |
|  | |
| file | stepsize\_adaptation.hpp [code] |
|  | |
| file | stepsize\_adapter.hpp [code] |
|  | |
| file | stepsize\_covar\_adapter.hpp [code] |
|  | |
| file | stepsize\_var\_adapter.hpp [code] |
|  | |
| file | var\_adaptation.hpp [code] |
|  | |
| file | windowed\_adaptation.hpp [code] |
|  | |


---

[ Stan Home Page ]

*© 2011–2014,
Stan Development Team.*
